# Difficulties in Determining the Pozzolanic Activity of Thermally Activated Lower-Grade Clays

**DOI:** 10.3390/ma17205093

**Published:** 2024-10-18

**Authors:** Kateřina Šádková, Vojtěch Pommer, Martin Keppert, Eva Vejmelková, Dana Koňáková

**Affiliations:** Department of Materials Engineering and Chemistry, Faculty of Civil Engineering, Czech Technical University in Prague, Thákurova 7, 166 29 Prague, Czech Republic; katerina.sadkova@fsv.cvut.cz (K.Š.); vojtech.pommer@fsv.cvut.cz (V.P.); martin.keppert@fsv.cvut.cz (M.K.); eva.vejmelkova@fsv.cvut.cz (E.V.)

**Keywords:** lower-grade clays, calcination, thermal activation, pozzolanic activity

## Abstract

Thermally activated clays (TACs) have been identified as possible supplementary cementitious materials (SCMs). To find a suitable clay and to optimise the activation process, it is necessary to determine its pozzolanic activity. However, the nature of clays is different from that of conventional SCMs. Therefore, the results of commonly used methods may differ; in some cases, they can even be misrepresented and misleading. This article aims to assess their applicability to TAC. Four direct and four indirect methods were compared by determination of the pozzolanic activity of three different clays calcined at varying temperatures. The isothermal calorimetry with lime combined with the mechanical strength’s development was identified as an ideal combination. Contrarily, the lime saturation test was inapplicable. For the Frattini method, it was found to be beneficial to assess the change in activity due to the thermal treatment rather than the strict comparison with a calcium hydroxide saturation curve.

## 1. Introduction

Due to the heavy environmental burden caused by Portland clinker production at high temperatures, other materials are currently being considered to replace the clinker, at least partially. Such materials are called supplementary cementitious materials (SCMs). To be used as an SCM, a material must demonstrate pozzolanic activity. In general, it means that it must contain compounds that react well with calcium hydroxide to produce C-S-H (calcium silicate hydrate) [1]. Currently, the most frequently used SCMs include microsilica [2,3,4,5], blast furnace slag [6,7], and fly ash [8,9,10]. In addition to saving energy-consuming and CO_2_-emitting clinker, SCMs can potentially improve the properties of the resulting composite. They positively affect the workability of concrete, increase its resistance to external influences, and, due to the later onset of pozzolanic reaction, the long-term mechanical properties of these composites are generally better than those of composites containing only cement [11,12,13].

Therefore, it is clear that to use SCMs in concrete optimally, it is essential to determine the pozzolanic activity with the greatest possible accuracy. Although it is clear what the pozzolanic activity means (the ability of a material to react with calcium hydroxide to form hydration products), it is not clear how to directly quantify it. Many types of tests have been developed for this purpose. One possible classification of tests assessing pozzolanic activity is into direct methods that measure the consumption of Ca(OH)_2_ by the pozzolanic reaction (Frattini test, lime saturation test, XRD, thermogravimetry) and indirect methods that measure the change in properties related to the pozzolanic reaction (strength activity index, pH reduction, electrical conductivity, conductive calorimetry). It is helpful to compare results of indirect methods with the results of direct methods to confirm that a pozzolanic reaction has occurred [14,15,16,17].

The most commonly used direct method is the Frattini test, which can accurately define the pozzolanic activity of blended Portland cement by measuring the consumption of Ca(OH)_2_ released during cement hydration. This test is susceptible to the chemical composition of the pozzolan used, especially the amount of alumina present [18,19,20]. The lime saturation test (or saturated lime test) has a similar principle; the pozzolan is mixed with a saturated lime solution (slaked lime; Ca(OH)_2_), and the amount of residual dissolved calcium is measured to determine the amount of lime bound by the pozzolan [14,21]. Another very effective direct method used to monitor pozzolanic activity is the isothermal calorimetry (IC) test, which monitors the amount of heat released during the pozzolanic reaction [17,22,23] or the test tracking the amount of binding water released during the pozzolanic reaction [24].

The most common indirect method called the strength activity index (SAI) correlates very well with the Frattini test [14,25,26,27]. With this method, the effect of the pozzolanic reaction on the strengthening of the cement matrix can be determined using the measured compressive strength. The mechanical strength depends more on the microstructure of the hydration products than on the amount of reacted Ca(OH)_2_. However, the results of this type of test are highly dependent on the amount of water added to the mixture [14,28,29,30]. The electrical conductivity test is one of the rapid indirect tests based on measuring the electrical conductivity of the suspension of pozzolan in a saturated lime solution [31,32,33]. The results of different tests have been analysed for different types of SCMs [14,34].

The majority of currently used SCMs originate as industrial and energy by-products. However, these materials are becoming increasingly scarce at least in some territories because coal energy and steel production have declined and use more environmentally friendly technology with lower amounts of waste. Another problem may be the need to import these materials from far away, which increases their purchase price [35,36,37]. Therefore, the ideal solution would be to find some local source of natural material that could be used as a substitute in cement composites. An example of such a material is clay containing kaolinite. The clays themselves are considered to be materials with low pozzolanic activity; but, when processed at 550–900 °C [38,39,40,41], the argillic minerals are dehydroxylated to form amorphous aluminosilicate phases (Al_2_O_3_·2SiO_2_—AS_2_, Al_2_O_3_·4SiO_2_—AS_4_), which are the carriers of the pozzolanic activity. The resulting product is called thermally activated clay (TAC) [42,43,44]. The most commonly used clay is indisputably kaolinite, which gives rise to metakaolin. Other lower-grade clays can be used for the production of pozzolanic material.

Based on many research studies, it is clear that the thermal activation process of clays is significantly influenced by the heating rate, time, and cooling conditions in addition to the temperature chosen [45,46,47]. The optimum heat treatment for activating different clays is assessed by measuring the pozzolanic activity of the calcined material. An important factor when comparing different methods is the time and temperature of treatment of the samples under investigation. For example, the Frattini test is specified for eight days at 40 °C, and the SAI test is established for 28 days at 23 °C (BS 3892 [48], EN 196-5 [49], and ASTM C311 [50]), while the saturated lime method can be performed at any time as there is no need to wait for the cement hydration process to complete [14]. The weight ratio of lime to pozzolan also dramatically influences the comparison of methods detecting pozzolanic activity. This ratio should be as similar as possible for the tests to make them more likely to be comparable [14].

Several experiments have been performed to compare methods for determining pozzolanic activity in different SCM types [25,51,52,53]. However, as it will be demonstrated in this paper, results, or more precisely methods, are not generally transferable to the investigation of TAC pozzolanic activity. Therefore, the main aims of this study are to compare different methods for determining pozzolanic activity and to identify those that can most accurately describe the pozzolanic activity of TACs. Among the wide range of methods, the Frattini test, lime saturation test, isothermal calorimetry, X-ray diffraction (XRD), mechanical strength, pH of cement pastes, strength activity index, and determination of specific surface area were selected. Regarding the used clays, three varying raw materials, different in phase composition, were chosen for this study. Thermal activation was performed at four different temperatures, specifically at 500 °C, 550 °C, 600 °C, and 650 °C. In addition to those TACs, the raw clays without temperature treatment were examined.

## 2. Experimental Methods

### 2.1. Raw Material Characterisation

The X-ray fluorescence spectrometry method (ARL Quant’X EDXRF spectrophotometer (Thermo ScientificTM, Waltham, MA, USA)) was used to determine the chemical composition of the investigated clays. UNIQANT 4 (Thermo ScientificTM, Waltham, MA, USA) software was used to evaluate the data.

The phase analysis of the raw clays was determined by XRD using a PANalytical Aeris diffractometer (Malvern Panalytical, Worcestershire, UK) equipped with a conventional X-ray tube (CoKα radiation, 40 kV, 30 mA, line focus). For data evaluation, Profex 5.2.4 software (Nicola Döbelin, Solothurn, Switzerland) [49] was used.

The transformation of minerals during the heating was also investigated by simultaneous thermal analysis (STA), consisting of differential scanning calorimetry (DSC) and thermogravimetry (TG) using a LABSYS EVO DTA/DSC device (SETARAM Inc., Caluire, France). A total of 60 mg of clay was used. The heating rate was 10 °C min^−1^ in an argon atmosphere with a flow rate of 40 mL min^−1^.

The granulometry of clays before firing was determined by the laser diffraction method (Bettersizer S3 Plus device, Meritics Ltd., Leighton Buzzard, UK). This instrument combines laser diffraction and dynamic image analysis.

### 2.2. Direct Methods for Pozzolanic Activity Assessment

#### 2.2.1. Frattini Test

The procedure specified in EN 196-5 [49] was used. A total of 20 g of test samples was prepared consisting of 80% CEM-I and 20% of the test pozzolan and mixed with 100 mL of distilled water. After preparation, samples were shaken for 8 days in a sealed plastic bottle at 40 °C. After 8 days, samples were vacuum filtered. The filtrate was analysed for [OH^−^] by titration against dilute HCl with methyl orange indicator and for [Ca^2+^] by pH adjustment to 12.5, followed by titration with 0.03 mol.L^−1^ EDTA solution using Patton and Reeder’s indicator (Sigma-Aldrich, St. Louis, MO, USA). The Ca^2+^ and OH^−^ contents of the resulting filtrate were compared with the solubility curve of Ca(OH)_2_ in an alkaline solution at the same temperature.

#### 2.2.2. Lime Saturation Test

The lime saturation test was performed by mixing 1 g of pozzolan with 100 mL of saturated Ca(OH)_2_ solution; the slurry was shaken by an orbital shaker (120 rpm) at 40 °C. Subsequently, after 24, 72, and 168 h, the suspension was filtered and the solution was analysed in the same way as in the Frattini test (EN 196-5) [49]. The results were expressed as % of Ca(OH)_2_ fixed by pozzolan.

#### 2.2.3. Isothermal Calorimetry

Two sets of isothermal calorimetry experiments were performed with Portland cement CEM I 42.5R and with lime hydrate (solid Ca(OH)_2_). Samples in the first set were consistent with those described in Section 2.3.1; the pastes contained 90% of CEM I and 10% of pozzolan, *w*/*c* = 0.3. The measurement was performed at 20 °C (EN 196-11 [54]); the used device was an 8-channel TAM Air calorimeter (TA Instruments, New Castle, DE, USA). The second set of experiments was performed on mixtures containing 75% of Ca(OH)_2_ and 25% of pozzolan at 40 °C. This test was inspired by ASTM Standard C1897 [55] and performed with the help of the same calorimeter. In order to distinguish the contribution of pozzolanic reaction to the entire measured heat flow, the reference sample containing fine silica sand (instead of calcined clay) was investigated as well.

#### 2.2.4. XRD Method

For the determination of amorphisation degree, the clays were analysed by XRD. As with the raw clay measurements, a PANalytical Aeris diffractometer equipped with a conventional X-ray tube (CoKα radiation, 40 kV, 30 mA, line focus) was used for the measurements. Apart from the samples with clays themselves, the second measurement with the internal standard was prepared for quantification of the amorphous matter. Namely, highly crystalline zincite ZnO was employed in the amount of 20%. Analysis of XRD patterns was performed with the help of Profex software [56] based on Rietveld refinement.

### 2.3. Indirect Methods for Pozzolanic Activity Assessment

#### 2.3.1. Mechanical Parameters

Cement pastes with 10% clay substitution were produced to measure the mechanical properties. The 10% replacement level was set based on the general assessment of pozzolanic materials. In general, there was no optimal amount for all pozzolanic materials. For example, silica fume has an optimal dosage of about 5–15% [57,58,59]; in the case of fly ash, the optimum is between 10 and 30% [58,59]; and metakaolin can be dosed up to 10–20% [58,59]. From the area of less conventional materials, it can be named recycled water treatment sludge whose dosage should be 10% [60], paper mill ash with an optimum of about 10–20% [61], or silica glass powder with 12.5% [62]. On that account, it was decided to examine 10%, which fell within the scope of optimal dosage for SCMs. The reference sample contained cement CEM I 42.5R as in the case of isothermal calorimetry. The used *w*/*c* ratio was 0.3. A total of 3 beams of 100 × 20 × 20 mm^3^ were made; smaller dimensions were designed in order to prevent cracks caused by autogenous shrinkage. Bending and compressive strengths were determined according to the standard procedure [63]. The bending strength was measured on a three-point scheme with a span length of 80 mm on an MTS 100 loading device. The compressive strength was measured on the remaining beam fragments on the EU40 loading device. These properties were examined at 28 and 180 days.

#### 2.3.2. pH Value

After the destructive measurement of mechanical strengths, the pH was determined on the crushed and grained samples according to BS 7755-3.2 [64]. The suspension, in a 5:1 ratio of liquid (deionised water) to solid, was stirred for 5 min and then allowed to settle for 1 h and 24 h. The pH value was measured on a pH meter 4 times. The average values of the measurements were plotted on a graph.

#### 2.3.3. Strength Activity Index (SAI)

The progress of the test conformed to the standard [65]. The compressive strength to determine SAI was measured on samples composed of the binder/aggregates ratio 1:3, and the *w*/*c* coefficient was 0.5. The reference sample contained cement, and the others contained blended binders with 20% of TAC replacement. Silica sand in 3 gradings (standard sand PG1–PG3) was used as aggregates. The water dosage used was according to the standard; *w*/*c* ratio of 0.5. After mixing, three beams of 160 × 40 × 40 mm^3^ were formed and allowed to harden in lime water. After curing at 7, 28, 90, and 180 days, the EU40 loading device was again used, with the compressive steel plates measuring 40 × 40 mm^2^. From the results, the SAI was determined as the ratio between the compressive strength of the reference and TAC-containing specimen according to standards [49,66,67].

#### 2.3.4. Specific Surface Area

The air permeability method and the automatic Blain instrument UTEST UTCM-0280 (UTEST material testing equipment, Ankara, Turkey) were used to determine the specific surface area of thermally activated clays, which have a significant impact on the pozzolanic activity as well [42]. The procedure was carried out according to the standard [68] and was based on comparative methods.

## 3. Studied Materials

### 3.1. Raw Materials and Their Characterisation

**Figure 1:**
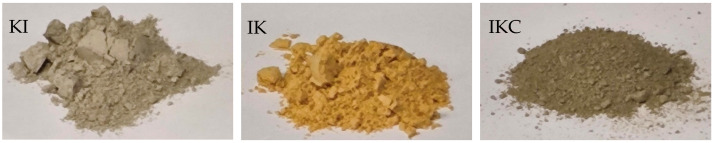
Studied clays.

**Figure 2:**
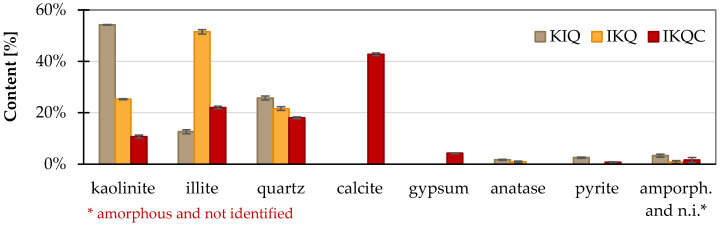
Phase composition of clays (XRD).

**Figure 3:**
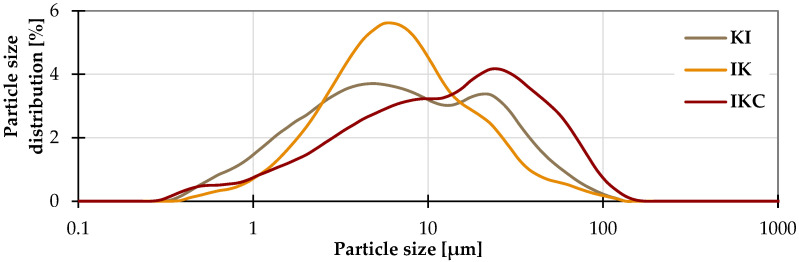
Granulometry of clays.

Clays from three different sources in the Czech Republic were used in the research (Figure 1). The clay KI is produced by the company Keramost a.s (Most, Czech Republic), clay IK was acquired from the company LB Minerals s.r.o. (Horní Bříza, Czech Republic), and the last material, denoted IKC, was brick soil from the company HELUZ cihlářský průmysl a.s. (Dolní Bukovsko, Czech Republic) The labels reflect the main specific minerals (K kaolinite, I illite, C calcite). More information about the clays is given in Table 1. Their chemical composition is in Table 2, and the phase proportions are summarised in Figure 2. The KI clay contains mostly kaolinite and a lower amount of illite and quartz. The IK clay has the reverse ratio of illite and kaolinite. The last IKC clay has a more diverse composition with significant amounts of calcite. Concerning the amorphous matter, all clays were found to be primarily crystalline with only a trace amount of amorphous phases. Studied clays slightly differ also from the point of view of the granulometry (Figure 3) where the clay IKC had somehow coarser grains, what is typical for brick soils. Regarding the characteristic values (Table 1), it is visible that all clays had equal D10 (10% of particles had a lower dimension than this size). D50 (median particle size) and D90 (90% of particles are smaller than this size) were about twice as high in the case of IKC compared to the other clays. Conversely, clay IK reached the lowest value of D90, showing the narrowest particle size distribution.

### 3.2. Thermal Activation

**Figure 4:**
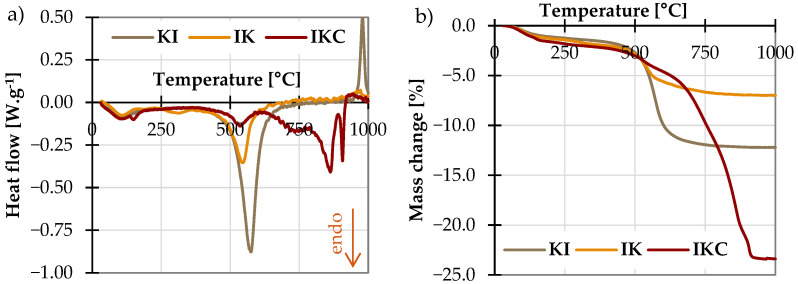
Simultaneous thermal analysis of clays: (**a**) DSC and (**b**) TG.

The results of simultaneous thermal analysis of studied raw clays are shown in Figure 4. Almost all reactions taking place in the studied clays were endothermic. It is visible that in the first dehydration range up to about 200 °C curves are almost equal, with only one exception of IKC. This clay showed another peak at about 150 °C, which corresponds to dehydration of gypsum. However, the most important for the need of this study was the dehydroxylation area, which took place in a range of 500–650 °C. In this area, clay minerals lose their inner bounded water, specifically hydroxyl groups (OH^−^), which are removed from the inner crystal structure. This consequently leads to the collapse of the originally layered structure and the amorphisation of clays. Based on this observation, temperatures for thermal treatment were selected within this range with a step of 50 °C; namely, the clays were exposed to 500 °C, 550 °C, 600 °C, and 650 °C. Thermal activation took place in a top cover electric furnace with a temperature rate of 10 °C min^−1^ and duration for 3 h. After firing, the clays were allowed to cool spontaneously.

To complement the description of the clay performance, the TG/DSC curves of sample IKC also feature the CaCO_3_ thermal decomposition above 600 °C; the broad temperature range, where the decomposition takes place, indicates that there is not only calcite (detected by XRD) but probably also some roentgen-amorphous species of CaCO_3_ [69]. The next sharp and narrow peak at 906 °C corresponds to the decomposition of anhydrite (originating in gypsum observed on the XRD, Figure 2) [70]. In all cases, the only exothermic peaks were observed over 950 °C; this reaction corresponds to the crystallisation of Al-spinel [71] and was more profound in the case of clay KI with the highest amount of kaolinite.

## 4. Results and Discussion

All experiments were carried out not only on thermally activated clays, which were marked by their label and activation temperature, but also on raw materials to show the effect of calcination.

### 4.1. Direct Methods

#### 4.1.1. Frattini Test

**Figure 5:**
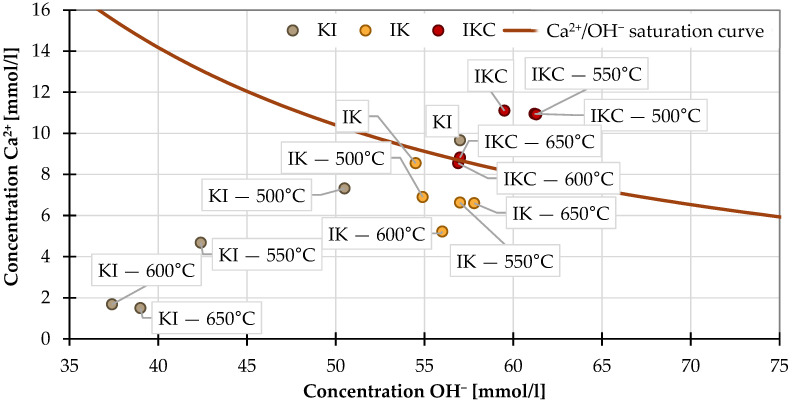
Frattini test results.

The results of the Frattini test are depicted in Figure 5. The Frattini test is classified as positive—material has a pozzolanic activity—when its “point” falls below the solubility curve of lime, more specifically below Ca^2+^/OH^−^ saturation curve (i.e., material is fixing Ca^2+^ ions from slurry and creates C-S-H products). The raw clays IK and KI are found very close to the curve, while IKC is above. The calcination of IKC did not cause significant “movement” below the curve, which means that—in terms of this test—this material is not fixing Ca^2+^ and does not have pozzolanic activity regardless of the calcination temperature. One problematic aspect is the higher content of calcite in the clay IKC, specifically the higher content of Ca^2+^ in the material itself, which can somewhat misload the reached results. On the other hand, the KI samples transferred with increasing calcination temperature well below the curve that implies high pozzolanic activity; specifically, the KI 600 and KI 650 samples reached very low Ca^2+^ content in filtrate. The points of IK samples are found close to each other, slightly below the curve, regardless of the calcination temperature; this indicates slight pozzolanic activity.

It is striking, however, that in this test, certain pozzolanic activity was also measured in unfired clay IK since even raw clay can absorb Ca^2+^ to some extent. In the case of this type of test, it is therefore advisable to examine the clay in its raw state and to observe the evolution of its pozzolanic activity. Rather than assess the position related to the calcium isotherm curve, consider the switch of the position related to unfired clay. Another possibility is to compare the Frattini test performed on activated clays with another method.

#### 4.1.2. Lime Saturation Test

**Figure 6:**
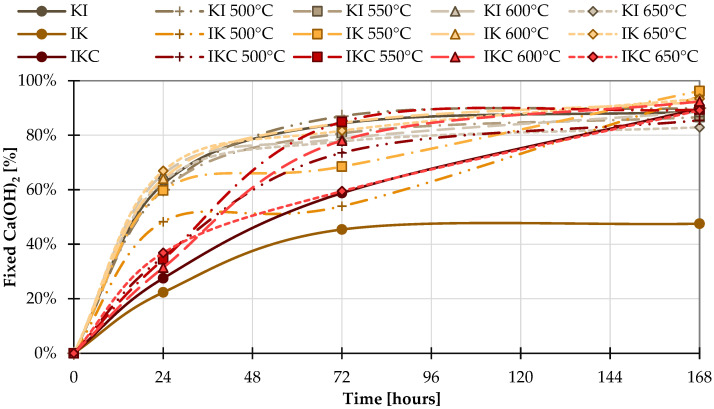
Lime saturation test results.

The resulting lime saturation test graph (Figure 6) shows the amount of Ca(OH)_2_ bound over a certain period. The bigger the amount of fixed Ca(OH)_2_ is, the higher the pozzolanic activity material shows. However, the results of clay KI confirmed that this clay itself in its raw state has a high ability to fix Ca^2+^. Moreover, firing temperature has minimal effect on the measured pozzolanic activity of KI clays. After only 72 h, these clays approached or exceeded 80% bound Ca(OH)_2_ regardless of the heat treatment. In contrast, IK clay showed slightly lower pozzolanic activity and slower ability to fix Ca^2+^. After 72 h, only IK clays fired at 600 and 650 °C reached a Ca(OH)_2_ uptake value of 80%. However, after 168 h, these clays reached the highest value of pozzolanic activity. The sample fired at 550 °C was the best, consuming almost 100% of available Ca(OH)_2_. In the case of the lime saturation test, the IKC clay also came out with pozzolanic activity. After 72 h, only the clay fired at 550 °C exceeded 80%, followed by the one exposed to 600 °C, which reached almost 80%. However, after 168 h, the amount of fixed Ca(OH)_2_ of all IKC clays was comparable to the other samples.

Overall, however, it is clear that this type of test is not applicable for determining the pozzolanic activity of calcined clays, as Donatello also notes in his article [14]. One reason is that in this test, all clays reach almost the same pozzolanic activity after 168 h, and the other reason is the manifestation of pozzolanic activity even in unfired clays. This phenomenon is particularly evident in the KI and IKC clays, where even the raw clay has bound the same amount of Ca(OH)_2_ as the fired clay after 168 h.

#### 4.1.3. Isothermal Calorimetry

**Figure 7:**
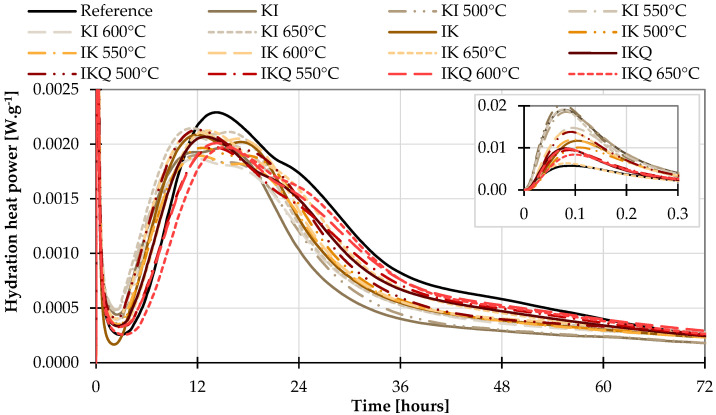
Isothermal calorimetry with cement.

The results of isothermal calorimetry of the first set with cement are shown in Figure 7. It seems that clay utilisation contributes to the increased wetting and dissolving heat (a first peak at about 5 min, inset in Figure 7) and serves as a nucleus at the beginning of hydration, which becomes earlier compared to the pure cement (denoted as “Reference”).

On the other hand, the maximum evolved hydration heat power is somehow reduced due to the clay’s utilisation, which can be attributed to the lower amount of C_3_S present in the samples with TAC. After 80 h, the pozzolanic reaction became evident and observable as the mixtures with clays reached higher values of hydration heat power. Nevertheless, considering the accuracy of the measurement and the differences between particular activated clays, it is visible that the pozzolanic reaction of the C_3_S with water is so pronounced that it obscures the degree of pozzolanic activity of the clay under testing.

**Figure 8:**
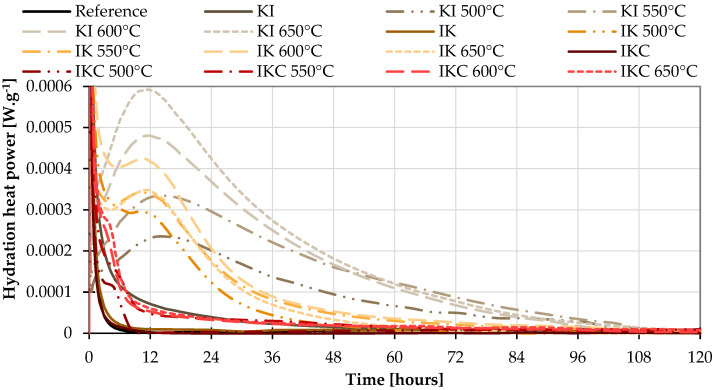
Isothermal calorimetry with lime.

As it was described in the test methods, the second set of isothermal calorimetry was performed in lime-based systems (Figure 8). It is indisputable that the released hydration heat power gives a very good indication of differences in pozzolanic activity of individual mixtures. More specifically, it identifies the ability of clay to react with Ca(OH)_2_. In this system, reference means the mixture of lime + sand. Similar to the cement systems, the first exothermic peak on each curve symbolises the so-called wetting heat (heat released immediately after mixing the material with water). The second peak indicates the heat released during the pozzolanic reaction. The more heat released, the more pozzolanic reaction took place. At first glance, it is apparent that the KI clays fired at 600 and 650 °C featured the best performance. In comparison, IK clays produced 50–60% less hydration heat. The pozzolanic activity of IKC is insignificant according to this test. The raw clays also did not generate any reaction heat. The results of the isothermal calorimetry correlate with the results of the Frattini test, but in this case, it is undisputed that the pozzolanic activity of the raw materials is zero. In addition, the time needed for this experiment is much lower, as the pozzolanic reaction is not delayed by the time up to the Portlandite creation as it is in Portland cement-based mixtures.

#### 4.1.4. XRD Method

**Figure 9:**
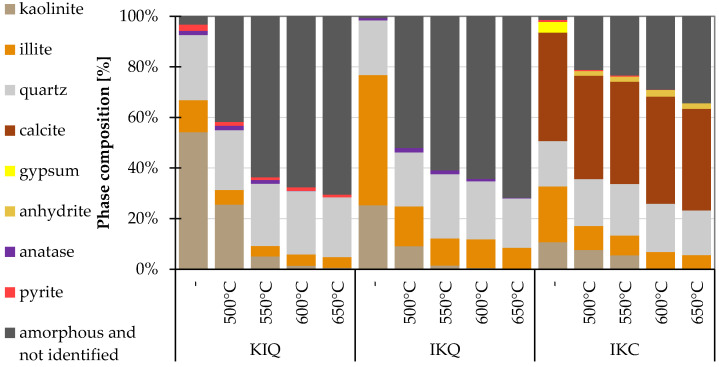
Phase composition of studied clays.

The results of the XRD analysis are shown in Figure 9. The thermal dehydroxylation of clay minerals results in a breakdown of their crystalline structure to roentgen-amorphous matter. The content of this amorphous portion in thermally activated clays was determined with the help of the internal standard. In this type of experiment, the more amorphous component in the mixture, the more pozzolanic reaction should occur. After firing at 600 and 650 °C, samples of KI and IK clays reached almost the same amount of the amorphous component. The IK clay after 650 °C was 2% higher than the KI clay fired at the same temperature. The IKC clay was also an order of magnitude 50% lower in this respect. It is obviously caused by the presence of high amounts of calcite. It can also be noted that the dehydroxylation of the clays examined occurs at 600 °C since almost no kaolinite remains in the mixtures after this temperature is reached. In contrast, some amount of illite is present in the mixtures even after exposure to the highest temperatures. The explanation of such phenomena can be searched in the presence of another phase. XRD patterns of illite and muscovite (both of these minerals belong to micas) are similar, and it is well-known that it is hard to distinguish them from each other only based on XRD. Taking into account muscovite dehydroxylate at a higher temperature of about 800 °C [69], it was assumed that illite was continuously decomposed at examined temperatures and the remaining phase was not pure illite but muscovite or some diffracting residuals of decomposed illite crystals. From that point of view, it would be probably more precise to call this phase hydromica than illite.

### 4.2. Indirect Methods

#### 4.2.1. Mechanical Properties of Cement Pastes

**Figure 10:**
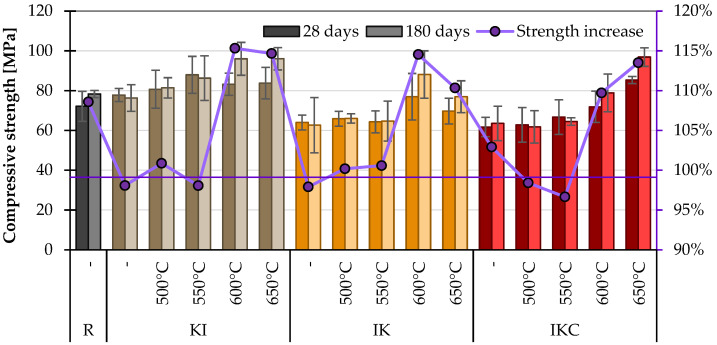
Compressive strengths on cement pastes.

In this chapter, the compressive strength of cement paste with 10% TAC content was studied. This replacement level was set considering the common dosage of convectional SCMs. The content that would provide more optimal strength would be chosen in practice for a specific TAC. It can be seen in Figure 10 that the KI clay calcined at 600 and 650 °C again achieved the highest compressive strengths and, hence, the highest pozzolanic activity (in terms of this method). Interestingly, both clays reached the same compressive strength of 96 MPa after 180 days, 19% higher than the reference mixture. Overall, the KI clay exceeded the compressive strength of the reference mix in all variations. The IK clay approached this strength after heat treatment at 600 °C, reaching a strength of 88.1 MPa after 180 days. Surprisingly, the paste containing IKC clay achieved the highest compressive strength after thermal activation at 650 °C—96.9 MPa. This was most likely because some of the binder (specifically grains of calcite) replaced the missing aggregate, and thus, the strength values of these materials—performing relatively poorly in other tests—were comparable to the others. Another reason can be sought in a further reaction between thermally activated clay and finer particles of CaCO_3_, which can be performed similarly as in the case of the limestone–metakaolin–cement system called LC^3^ [72,73].

It is also interesting to compare the percentage changes in strength development between 28 and 180 days. The curve in the x-plot shows the compressive strength development (CSD, counted as strength change during the time), which ranges from −2 to 15%. Clay itself in its raw state hurts mechanical strength [74,75]. It is caused by several aspects, but the main reason is that due to its hygroscopic ability, it undergoes volumetric changes, which leads to a disruption of a concrete/paste structure. It can be simply deduced that when the compressive strength in time increases equally or higher than the case of reference Portland cement, the clay shows pozzolanic activity. More universally, the material can be considered as a pozzolana active when the increase in compressive strength is higher than 10%. This means that all clays under study should be activated by temperature over 600 °C.

**Figure 11:**
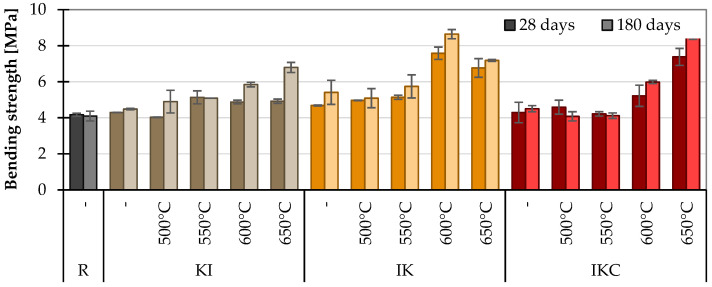
Bending strength on cement pastes.

The bending strengths in Figure 11 generally show similar tendencies as in the case of compressive strengths—positive impact of thermal activation. The only exceptions are the IK clay paste at 600 °C and the IKC clay paste at 650 °C, which somehow deviate from the values. Such a performance can be explained by a higher amount of crystal phases in these TACs, which can interlock together and thus help to inhibit the propagation of cracks. Nevertheless, it is worth mentioning that contrary to compressive strength, raw clays and not sufficiently activated clays (up to about 550 °C) still show equal or higher values as a reference for pure cement paste.

##### 4.2.2. pH Value of Cement Pastes

**Figure 12:**
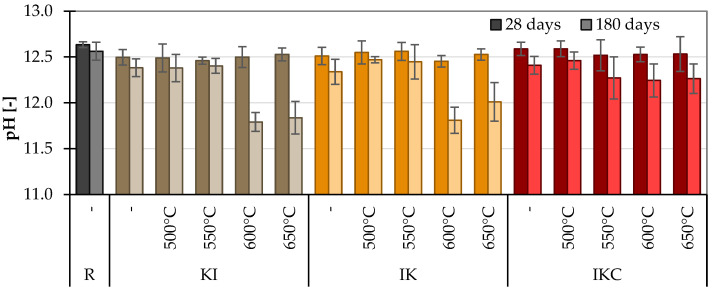
pH values of cement pastes.

The pH of a saturated solution of Ca(OH)_2_ is 12.5. The hydrated Portland cement (or its pore solution) very quickly reaches pH between 12.5 and 13, depending on the content of Na_2_O and K_2_O in clinker (virtually, the cement without any Na_2_O and K_2_O would have pH 12.5 being controlled just by Ca(OH)_2_). The “pH pozzolanic activity test” is based on the pH decrease in time caused by Ca(OH)_2_ consumption in a pozzolanic reaction. Higher pozzolanic activity of the used SCM would cause a faster reduction in pH (Figure 12) of the pore solution. Obviously, the pH of cementitious materials decreases also due to a carbonation—reaction of Ca(OH)_2_ with atmospheric CO_2_. The carbonation takes place from the material’s surface to the inner part. In this study, the pH was measured on the crushed samples of pastes after strength measurement. It means that the sample was homogenised: the surface parts were mixed with the inners, and the carbonated part of the material formed just a minor part of the specimen. Moreover, all samples were stored in the same conditions; thus, the level of their carbonation was the same, and the difference in pH may be thus attributed to the different pozzolanic activity of individual tested SCMs.

The results for 28 days are in fact equal in all materials, regardless of the kind of clay and activation temperature. Later, after 180 days of hydration, one already observes some changes. The difference between pH after 28 and 180 days of hydration (Figure 12) corresponds to the relatively slow onset of pozzolanic reaction in Portland cement-based systems. However, in the case of KI and IK activated at 600 and 650 °C, the remarkable decrease is caused by Ca(OH)_2_ consumption in the pozzolanic reaction. In the case of IKC, the difference between control and clay-containing pastes was less pronounced. Nevertheless, considering the results, this measurement can be employed for the pozzolanic activity confirmation but not for the assessment of convenient temperature as the differences between particular treatments are quite low. In addition, this approach for comparison of pozzolanic activity is rather time-consuming.

##### 4.2.3. Strength Activity Index

**Figure 13:**
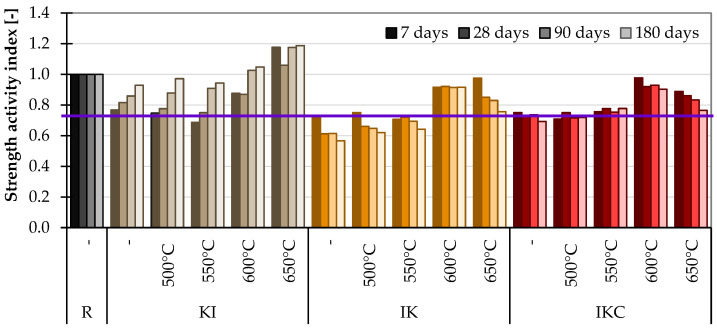
Strength activity index of cement composites.

For the SAI, 20% of the cement in the concrete was replaced by a clay alternative. According to the standard, the material is pozzolanic if the SAI exceeds 0.75 [67]. Looking at Figure 13, it is visible that such a condition is fulfilled in almost all cases of studied materials except for IK clays at the raw state and up to 550 °C. The samples exposed to 600 and 650 °C again reached the best values. The other two clay materials achieved better values after firing at 600 °C than at 650 °C. However, even in this test, care must be taken that unfired material comes out as having pozzolanic activity, as in the case of KI clay. On that account, it should be reasonable to either increase the limit of the minimal value of SAI (0.75) or again assess the impact of a thermal activation compared to the property of raw clay.

##### 4.2.4. Specific Surface Area

**Figure 14:**
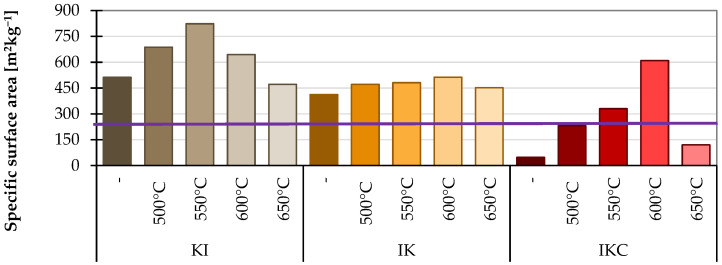
Specific surface area of examined clays.

The results of the last test can be found in Figure 14. The larger the specific surface area of the clay, the more reactive it should be. This experiment was added more for clarification or supplementation of clay performance than as a strict assessment of the pozzolanic activity. However, the specific surface area goes up due to the dehydroxylation. The collapse of the inner clay structure is accompanied by the creation of new pores (micro- or nano-scales) within the materials. Moreover, the reorganisation of the originally layered structure itself can lead to formation of more fragmented particles, which also contributes to the increase in specific surface area.

Due to the thermal activation, an undesirable agglomeration of grains can occur. Such a phenomenon leads to the decrease in pozzolanic activity and it can explain why a higher temperature of 650 °C is not always the better option despite having slightly higher amorphous content. Moreover, it proposed an option how to further increase the pozzolanic activity of TACs. According to the results, the finest clay is KI treated at 550 °C. The IK clay maintains a relatively constant value of specific surface area across all temperatures. Again, the worst values were achieved by the brick clay IKC. There is a sharp difference between the temperatures. The best result occurred at 600 °C, and then bigger sintering or agglomeration occurred, and this caused a rapid drop in specific surface areas. This test can determine if the reduced reactivity after exposure to temperatures higher than 600 °C is due to too-coarse granulometry and a small specific surface area. Common OPC has a specific surface area of about 300 m^2^kg^−1^, and the SCMs should reach an equal or preferably higher value.

### 4.3. Summary of Reached Results

In Table 3, the results of all methods are listed. For all methods, the three results that came out most reactive and the three results that came out least reactive are shown. The first row of the table shows the most reactive clay, and the last row shows the least reactive clay. At first glance, there is no doubt that the KI clay fired at 600 °C and 650 °C was the most reactive of the materials tested. The lime saturation test was the only test in which another clay came out as the most reactive. The KI 650 clay even ranked as the least reactive in this case. This is further evidence that this test is unsuitable for investigating the pozzolanic activity of thermally activated clays. Although, in many cases, the raw clays also seem to show a certain degree of reactivity, it is clear from the summary table that they were the lowest regarding pozzolanic activity. Mixtures containing IKC brick clay also showed very insignificant values.

In summary, there is no one ideal method that is best suited for determining the pozzolanic activity of thermally activated clays. It is always necessary to select a combination of several methods, and only on a base of multiple results can one decide which clay is the most reactive and which temperature is the most suitable. Isothermal calorimetry with lime combined with compressive strength development (CSD) or strength activity index (SAI) on 180-day-old samples emerged as the best combination from this study. The XRD method is suitable for a better understanding of pozzolanic activity based on the course of chemical reactions caused by exposure to high temperatures. The specific surface area method should be considered in all cases for searching for better reactivity or explaining lower reactivity of the clay under investigation after firing at a higher temperature. Contrarily, despite being used in many pieces of research, the lime saturation test was found to be inapplicable for TACs.

## 5. Conclusions

This paper aimed at applying methods used for the determination of the pozzolanic activity of thermally activated clays. Several direct and indirect methods were applied on three varying clays (in raw state, and thermally activated at 500 °C, 550 °C, 600 °C, and 650 °C). The results of the comparison of the methods for determining their pozzolanic activity can be summarised in the following points:

- When measuring the pozzolanic activity of thermally activated clay materials, it is advisable to include the raw material in the measurement. The result is then more comprehensive, and the development of the onset of pozzolanic activity can be deduced. Examining the raw material also checks the method’s suitability, as the raw material should show no or minimal pozzolanic activity.
- Especially in the case of the Frattini experiment, it is of great importance to also study raw clay. On the first view, this test proposed applicable results; however, due to the ability of raw clay to fix Ca^2+^, the results are distorted. Thus, more reasonable is to assess the shift of the TAC compared to a raw state than just consider if the sample is above/below the limit given by the saturation curve.
- The lime saturated test is unsuitable for investigating TAC reactivity, as the raw clays seemed to be reactive due to ionic exchange, and almost all clays reached the same Ca(OH)_2_ bound values after 168 h of testing. The most reactive clays, in this case, were completely different from all other tests.
- Isothermal calorimetry applied to samples containing lime instead of cement ranked among the most suitable types of tests used to determine pozzolanic activity. The raw clays were found to be non-reactive, and the amount of hydration heat released allowed the material’s reactivity to be reliably determined.
- The XRD method can serve as an excellent complementary direct method in this type of measurement, showing the reactivity of clays based on the amount of amorphous phase and the consumption of the clay minerals contained. However, it should be noted that phase quantification is somehow difficult in the case of clay minerals.
- Measurements of the mechanical properties of cement pastes containing TACs at one age are unreliable. In this case, more credible results proposed searching the strength development, which in the case of pozzolana should be over 10% per 180 days.
- SAI should be carried out on specimens aged for at least 28 days but more likely for longer. In addition, the limit of 0.75 for pozzolanic activity proof of TACs is insufficient because the raw clays can easily exceed this value. On that account, it should be higher, at least 0.85.
- Monitoring the specific surface area is somewhat inaccurate because it is affected by clay sintering. Still, as an additional measurement, it can be helpful for a more detailed understanding of the effect of grinding fineness on the reactivity of the clay under test.

There is no ideal single method for assessing the pozzolanic activity of calcined clays. It is advisable to individually select a combination of methods (preferably one direct method confirmed by an indirect one). In addition, it is advisable to evaluate the results not only for calcined clays but also to consider the performance relating to the raw non-calcined clay.

## Figures and Tables

**Table 1:** Physical properties of raw materials.

Label	Locality	Density [kg m^−3^]	Specific Surface Area [cm^2^ g^−1^]	D10 [µm]	D50 [µm]	D90 [µm]
KI	CZ—Brník	2520	5123	1.33	6.65	29.03
IK	CZ—Horní Bříza	2397	1038	1.99	6.45	21.08
IKC	CZ—Libochovice	2598	477	1.97	13.5	47.17

**Table 2:** Chemical composition of clays (XRF).

	LOI [wt.%]	Chemical Composition [wt.%]								
		SiO_2_	Al_2_O_3_	Fe_2_O_3_	CaO	K_2_O	MgO	TiO_2_	SO_3_	Na_2_O
KI	23.8	52.2	38.8	2.3	0.0	1.3	0.8	1.7	2.6	0.0
IK	13.7	52.8	33.7	4.8	0.1	5.2	1.4	1.2	0.0	0.4
IKC	26.9	36.8	14.3	4.4	36.6	3.1	2.4	0.8	1.3	0.0

**Table 3:** Summary of all methods.

Test		Age [Days]	Pozzolanic Activity					
			Best Results			Worst Results		
Direct methods	FT	8	KI 650	KI 600	KI 550	IKC 500	IKC 550	IKC
	LST	3	KI 500	IK 600	IKC 550	IKC	IK 500	IK
		7	IK 550	IK 500	IK 650	IKC 500	KI 650	IK
	IC—lime	5	KI 650	KI 600	KI 550	IKC 500	IK	IKC
	XRD	0	IK 650	KI 650	KI 600	KI	IKC	IK
Indirect methods	cement paste	28	KI 550	IKC 650	KI 650	IK	IKC 500	IKC
		180	IKC 650	KI 650	KI 600	IKC	IK	IKC 500
	CSD	180	KI 600	KI 650	IK 600	KI 550	IK	IKC 550
	pH	180	KI 600	IK 600	KI 650	IK 550	IKC 500	IK 500
	SAI	28	KI 650	IK 600	IKC 600	IK 550	IK 500	IK
		180	KI 650	KI 600	KI 500	IK 550	IK 500	IK
	SSA	0	KI 550	KI 500	KI 600	IKC 500	IKC 650	IKC

## Data Availability

All measured data and images are available upon request as well as any other detailed information about particular measurement processes.
